# A Novel Shunt Zigzag Double-Tap Low-Harmonic Multi-Pulse Rectifier Based on a Three-Stage Power Electronic Phase-Shifting Transformer

**DOI:** 10.3390/s24175564

**Published:** 2024-08-28

**Authors:** Xiuqing Mu, Xiaoqiang Chen, Qianxiao Liu, Ying Wang, Tun Bai, Leijiao Ge, Xiping Ma

**Affiliations:** 1 School of Automation and Electrical Engineering, Lanzhou Jiaotong University, Lanzhou 730070, China; 2 School of Electrical Automation and Information Engineering, Tianjin University, Tianjin 300072, China; 3 State Grid Gansu Electric Power Company Electric Power Science Research Institute, Lanzhou 730070, China

**Keywords:** three-stage power electronic phase-shifting transformer, double-tap, multi-pulse rectification, power factor correction, harmonic control

## Abstract

To solve the problem of the large size of traditional industrial frequency phase-shift transformers and the harmonic distortion of multi-pulse wave rectifier systems, this paper proposes a three-stage shunt zigzag power electronic phase-shift transformer based on a double-tap multi-pulse wave rectifier, which combines the power factor correction (PFC) converter with the voltage-type SPWM inverter circuit to form a power electronic converter to realize the frequency boost and power factor correction. Through AC–DC–AC conversion, the frequency of the three-phase AC input voltage is increased, the number of core and coil turns in the transformer is reduced to reduce the size of the phase-shifter transformer, a zigzag structure of the phase-shifter transformer is used to solve the unbalanced distribution of current between the diode bridges, and a passive harmonic suppression method on the DC side is used to generate a loop current by using a group of single-phase rectifier bridges to regulate the input line current of the phase-shifter transformer. The phase-shifted voltage is input into two three-phase diode rectifier bridges to rectify and supply power to the load. Simulation and semi-physical test results show that the proposed method reduces the total harmonic distortion (THD) value of the input current of the phase-shifted transformer to 7.17%, and the THD value of the grid-side input current is further reduced to 2.49%, which meets the harmonic standard and realizes the purpose of power factor correction as well as being more suitable for high-power applications.

## 1. Introduction

With the rapid development of energy interconnections and smart grids, the reliability, flexibility, and quality of power supply need to be of higher quality [1,2,3]. A multi-pulse rectifier (MPR) has the advantages of low complexity, high reliability, and high overload capacity, and it is widely used in high-power applications such as wind turbines, new energy generation, offshore wind power, and speed-regulated motors [4,5,6,7,8,9,10]. As one of the most important components of the multi-pulse wave rectifier system, the phase-shifting transformer has been widely studied by scholars for its large size, harmonic distortion, and system effects.

In order to solve the volume problem, studies [11,12] have proposed several autotransformers, but the existence of the AC and DC side of the electrical connection, affecting the safety of the system and autotransformers, is inconvenient for the regulation of the voltage, which is mainly used in non-isolated occasions, restricting the scope of its application.

In addition, to take into account the isolation of the transformer while reducing its size, one study [13,14,15] used single-phase high-frequency transformers for power conversion and transfer, which provides a novel method for the development of power electronic transformers based on AC–AC power conversion. Another study [16,17] proposed to apply two-stage-type power electronic transformers in MPR circuits to reduce the number of core and coil turns inside the phase-shifter transformer by increasing the frequency, which in turn reduces the volume of the phase-shifter transformer. Also, it guarantees the original power quality while reducing the volume of the phase-shifter transformer by one-third, but the harmonic distortion rate on the grid side is higher.

To address harmonic issues, references [18,19] proposed an active harmonic suppression method based on an active balanced inductor. When the DC-side circulating current is modulated to a standard triangular wave, harmonics in the grid-side input current can be minimized to the greatest extent. References [20,21,22] utilized passive harmonic suppression by employing passive auxiliary circuits on the DC side of the multi-pulse rectifier to increase the number of input current steps and output voltage pulses, thereby improving the power quality on both the input and output sides of the rectifier and achieving harmonic suppression effects.

However, as the number of phases in the transformer output increases, the winding structure becomes increasingly complex, leading to increased costs and manufacturing difficulty. Therefore, it is necessary to consider system input current harmonics while optimizing system volume, which is of great significance for MPR applications in high-power rectification systems.

Parallel multi-pulse rectifiers can increase the rectified output current. The zigzag structure can reduce the zero-sequence current in the circuit and solve the problem of uneven current distribution between diode bridges [23]. Power electronic transformers have a series of advantages, including voltage level conversion, electrical isolation, power regulation, and control [24].

Based on the above analysis, the system proposed in this paper reduces the size of the isolated phase-shifter transformer while utilizing a power factor correction (PFC) circuit for harmonic suppression at the grid side to meet the harmonic criteria. In this paper, the structure of the three-stage power electronic phase-shifting transformer (PEPT) MPR circuit and the optimal-turns ratio design of the double-tap are first analyzed in depth. The harmonic distortion rate of the input current is calculated and finally verified by the semi-physical platform and analyzed by comparative tests with different harmonic suppression methods.

## 2. Proposed Topology

**Figure 1:**
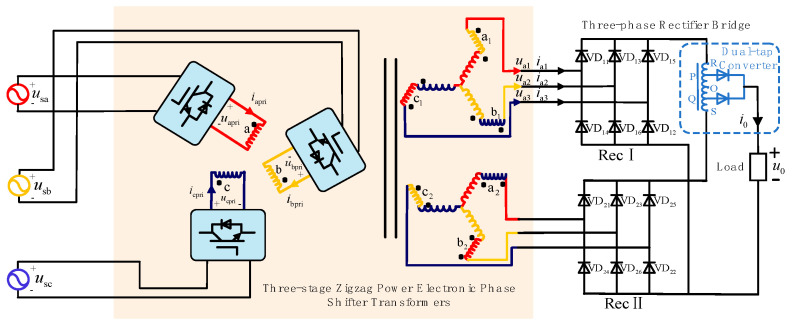
Topology of the shunt zigzag double-tap low-harmonic MPR circuit based on the three-stage PEPT.

Figure 1 shows the proposed shunt zigzag double-tap low-harmonic MPR circuit topology based on the three-stage PEPT. This topology consists of a three-phase power supply, a three-stage PEPT, a three-phase rectifier bridge, a dual-tap converter, and a load. The currents *i*_apri_, *i*_bpri_, and *i*_cpri_ are the input currents to the phase-shifting transformer; *i*_a1_, *i*_b1_, and *i*_c1_ are the input currents to Rec I; *i*_0_ and *u*_0_ are the load current and voltage, respectively. After the three-phase voltage (*u*_sa_, *u*_sb_, and *u*_sc_) is input to the three-stage PEPT, the three-phase high-frequency AC voltages *u*_apri_, *u*_bpri_, and *u*_cpri_ are obtained, which, after phase shifting by the shunt zigzag transformer, produce two sets of high-frequency three-phase voltages (Rec I comprises *u*_a1_, *u*_a2_, and *u*_a3_) directly connected to two sets of rectifier bridges. The DC current outputted by the two rectifier bridges is supplied to the load after being paralleled through the dual-tap converter. The instantaneous voltage differences generated between the rectifier bridges are absorbed by a balancing reactor.

For the theoretical analysis of Figure 1, the following assumptions are made: (1) The three-phase power source is ideal; (2) the system operates in the continuous conduction mode of inductor current; (3) the leakage inductance of the high-frequency phase-shifting transformer and dual-tap converter as well as the load are ignored; (4) all switches are considered ideal devices.

### 2.1. Three-Stage PEPT

The three-stage PEPT in Figure 1 consists of several power electronic devices and a high-frequency isolated phase-shifting transformer, wherein the power electronic devices comprise a PFC converter and a voltage-type SPWM inverter.

**Figure 2:**
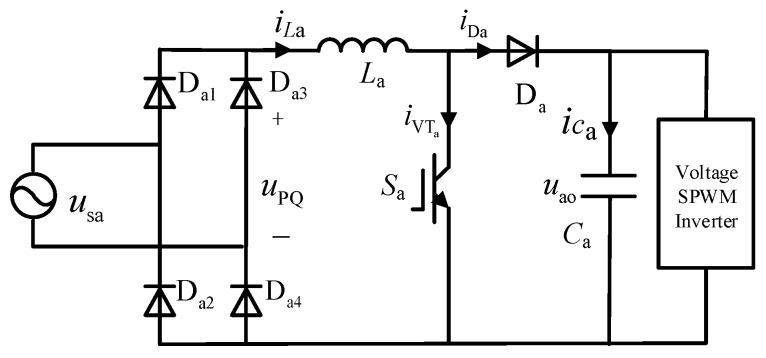
Topology of PFC converter.

Taking phase a as an example, the PFC converter consists of a single-phase rectifier bridge (bridged by D_a1_, D_a2_, D_a3_, and D_a4_) connected to a boost circuit (comprising inductor *L*_a_, switch S_a_, and diode D_a_), as shown in Figure 2.

**Figure 3:**
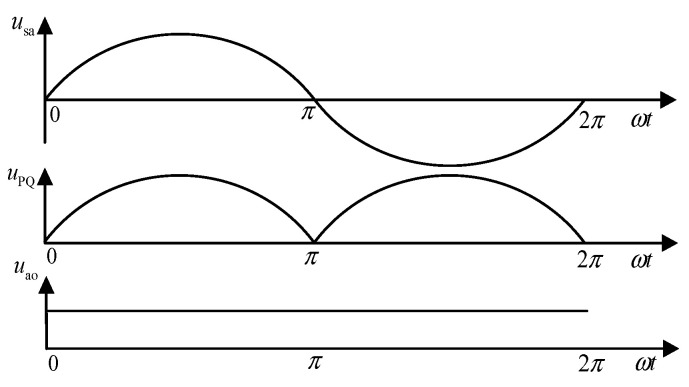
Voltage waveform before and after the PFC converter.

When the AC voltage passes through the single-phase rectifier bridge, it is converted from AC to DC, and the waveform changes from a sinusoidal wave to a DC mantou wave, as shown in Figure 3. *u*_PQ_ is the output voltage of the rectifier bridge, and *u*_ao_ is the output voltage of the boost circuit.

We assume the three-phase AC input voltage as below:

(1) $$\left\{ \begin{array}{l} U_{\mathrm{sa}}=\sqrt{2}E\sin\omega t\\ U_{\mathrm{sb}}=\sqrt{2}E\sin\left(\omega t-120^{\circ }\right)\\ U_{\mathrm{sc}}=\sqrt{2}E\sin\left(\omega t+120^{\circ }\right) \end{array}\right.$$

In Equation (1), *E* represents the effective phase voltage, and *ω* denotes the angular frequency of the three-phase AC power supply.

Therefore, taking phase a as an example, the voltage *u*_PQ_ after passing through the single-phase uncontrolled rectifier bridge can be obtained:

(2) $$u_{\mathrm{PQ}}=\sqrt{2}E\left|\sin\omega t\right|$$

**Figure 4:**
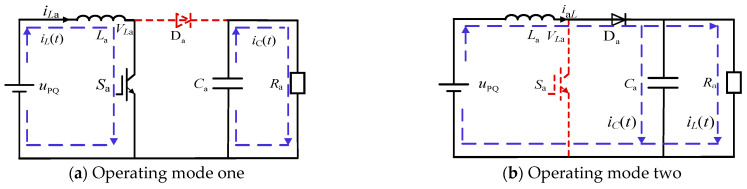
Two different operation modes of PFC converter.

The output load of the PFC converter is equivalently represented as *R*_a_. The switching transistor of the boost circuit operates in two different modes corresponding to the conduction and cutoff conditions, as illustrated in Figure 4a,b.

Under operating mode one, when *S*_a_ is conducting, *u*_PQ_ charges the inductor *L*_a_, and the capacitor *C*_a_ discharges to the equivalent resistor *R*_a_. The voltage across the inductor *V_L_*_a1_ is as follows:

(3) $$V_{\mathrm{La}1}(t)=L_{a}\frac{di_{\mathrm{La}}(t)}{dt}$$

Under operating mode two, when *S*_a_ is turned off, both *u*_PQ_ and inductor *L*_a_ charge the capacitor *C*_a_ while discharging to the resistor *R*_a_ simultaneously. At this time, the voltage across the inductor *V*_La2_ satisfies the following:

(4) $$V_{\mathrm{La}2}(t)=L_{a}\frac{di_{\mathrm{La}}(t)}{dt}=u_{\mathrm{PQ}}(t)-u_{\mathrm{ao}}(t)$$

When the switching frequency of *S*_a_ is much higher than the frequency of the power supply cycle, it can be assumed that the charge and discharge quantities of the inductor are the same in both operating modes one and two. Therefore, the linear equation for the inductor voltage over one cycle is as follows:

(5) $$\int_{0}^{{\mathrm{DT}}_{\mathrm{PWM}}}v_{\mathrm{La}1}(t)d(t)+\int_{{\mathrm{DT}}_{\mathrm{PWM}}}^{T_{\mathrm{PWM}}}v_{\mathrm{La}2}(t)d(t)=\sqrt{2}E\left|\sin(\omega t)\right|T_{\mathrm{PWM}}+(1-D)u_{\mathrm{ao}}T_{\mathrm{PWM}}=0$$

In Equation (5), T_PWM_ represents the PWM switching period, and *D* is the duty cycle of the inductor voltage input to output. 

From Equation (5), the expression for the duty cycle *D* is the following:

(6) $$D(t)=1-\frac{\sqrt{2}E\left|\sin(\omega t)\right|}{u_{\mathrm{ao}}}$$

When the duty cycle satisfies Equation (6), the inductor current remains continuous, allowing it to follow the changes in *u*_PQ_, thus making the input current more sinusoidal and achieving power factor correction.

**Figure 5:**
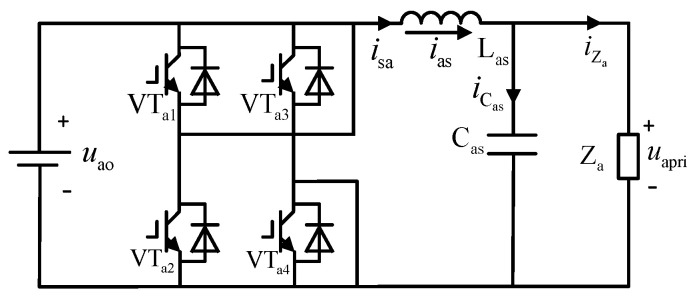
Topology of voltage-type SPWM inverter.

In the inverter circuit, the voltage-type SPWM inverter consists of a single-phase full bridge inverter circuit (bridged by VT_a1_, VT_a2_, VT_a3_, and VT_a4_) and an *LC* filter (comprising *L*_as_ inductor and *C*_as_ capacitor) with topology as shown in Figure 5.

**Figure 6:**
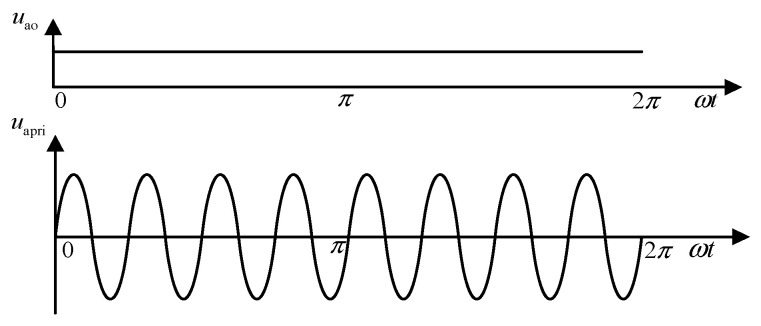
Voltage waveforms before and after the inverter circuit.

After rectification by the PFC, the DC voltage is inverted into a high-frequency sine wave. *u*_ao_ and *u*_apri_ are the output voltages of the boost circuit and the *LC* filter, respectively, with the waveforms shown in Figure 6.

**Figure 7:**
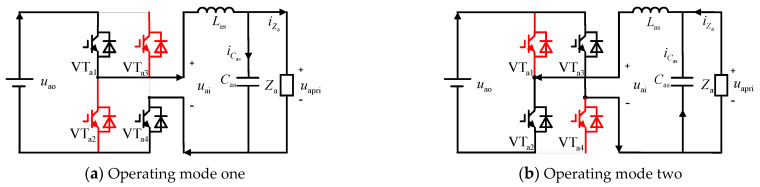
Two operation modes of voltage-type SPWM inverter.

The inverter also has two operating modes, as shown in Figure 7a,b. In operating mode one, VT_a1_ and VT_a4_ are conducting, while VT_a2_ and VT_a3_ are off. Here, the output voltage is equal to the input voltage. In operating mode two, the situation is completely reversed: VT_a2_ and VT_a3_ are conducting, while VT_a1_ and VT_a4_ are off, resulting in a negative output voltage relative to the input voltage.

Assuming the modulation index of the inverter is *M*, the output voltage *u*_ai_ of the inverter is as below:

(7) $$u_{\mathrm{ai}}=Mu_{\mathrm{ao}}$$

Based on the equations for inductor current and capacitor voltage, the current flowing through the capacitor *C*_as_ and the voltage across the inductor *L*_as_ can be determined as follows:

(8) $$\left\{ \begin{array}{l} L_{\mathrm{as}}\frac{di_{L_{\mathrm{as}}}(t)}{dt}=u_{\mathrm{ai}}(t)-u_{C_{\mathrm{as}}}(t)=u_{L_{\mathrm{as}}}\\ C_{\mathrm{as}}\frac{du_{\mathrm{apri}}(t)}{dt}=i_{L_{\mathrm{as}}}(t)-i_{Z_{a}}(t)=i_{C_{\mathrm{as}}} \end{array}\right.$$

When the impedance of the *LC* filter is zero, the *LC* filter can be regarded as a voltage source. By simultaneously solving Equations (2), (6), and (7), the input voltage *U*_apri_, *U*_bpri_, and *U*_cpri_ of the high-frequency phase-shifting transformer can be obtained as given:

(9) $$\left\{ \begin{array}{l} U_{\mathrm{apri}}=\frac{\sqrt{2}EM}{1-D}\sin\varphi \omega t\\ U_{\mathrm{bpri}}=\frac{\sqrt{2}EM}{1-D}\sin(\varphi \omega t-\frac{2\pi }{3})\\ U_{\mathrm{cpri}}=\frac{\sqrt{2}EM}{1-D}\sin(\varphi \omega t+\frac{2\pi }{3}) \end{array}\right.$$

In Equation (9), *φω* represents the total number of voltage oscillations within one period, and *φ* is the multiplication factor to increase the frequency.

### 2.2. Specific Control Strategy

**Figure 8:**
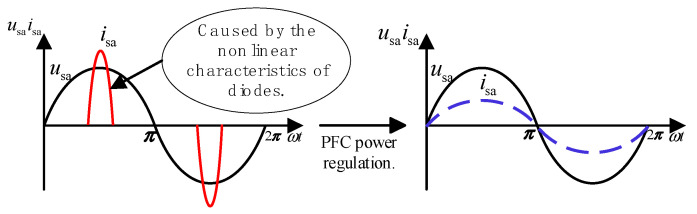
Waveform before/after PFC converter regulation.

As shown in Figure 8, before the addition of the PFC converter, the system input voltage is sinusoidal. Due to the nonlinear characteristics of the single-phase diodes in the single-phase rectifier bridge, the system’s input current is severely distorted, reducing the system power factor and causing energy waste.

**Figure 9:**
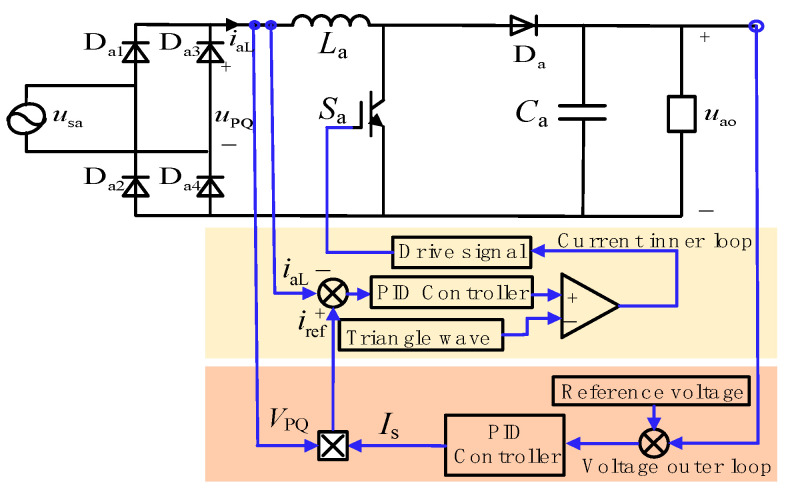
The PFC converter control.

In continuous conduction mode, the PFC converter control is shown in Figure 9. The outer voltage loop ensures stability of the DC bus voltage on the output side, while the inner current loop ensures that the input inductor current exhibits a sinusoidal envelope. The outer voltage loop provides amplitude information *V*_PQ_ for the current reference signal *I*_s_ of the inner current loop while also adjusting the boost converter output voltage. The inner current loop makes the inductor current *i*_aL_ follow the current reference signal *i*_ref_, achieving power factor correction.

**Figure 10:**
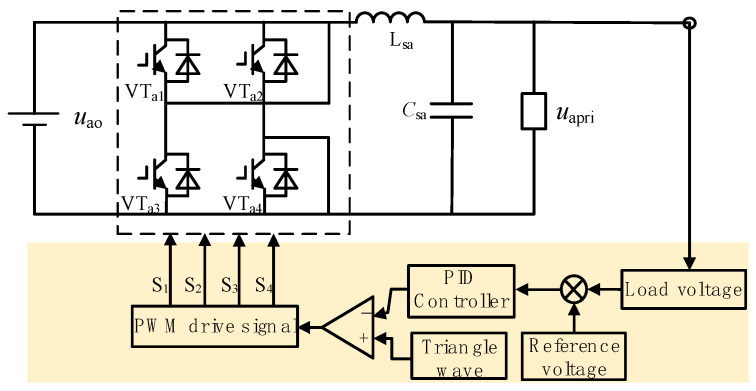
The SPWM inverter control.

The control of the SPWM inverter is illustrated in Figure 10. The output voltage *u*_apri_ of the *LC* filter is taken as the controlled object. The error between its measured value and the given sinusoidal reference signal is calculated. This error signal is dynamically adjusted by a PID controller in real-time, generating control signals S_1_, S_2_, S_3_, and S_4_. These signals ensure that the inverter output voltage follows the sinusoidal reference signal and maintains a constant output voltage magnitude.

### 2.3. Shunt Zigzag Phase-Shifting Transformer

**Figure 11:**
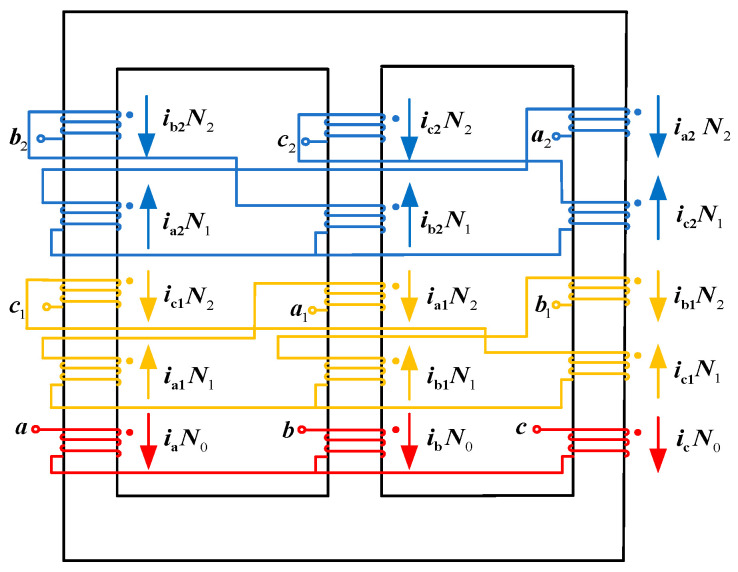
Zigzag-type isolation transformer winding structure.

As shown in Figure 11, our research team investigated a winding structure based on a zigzag phase-shifting transformer [8,9].

According to Faraday’s Law of Electromagnetic Induction, the higher the operating frequency of the transformer, the faster the rate of change of magnetic flux, and the greater the induced potential; in the need to generate the same potential occasions, medium- and high-frequency phase-shifted transformers require much fewer core and coil turns than the industrial frequency phase-shifted transformers. From the study [25], the change in volume of steel–silicon transformers at different frequencies is known, and the change in volume with frequency is less pronounced at 400 Hz, so this study also chose to analyze at this frequency.

In Figure 11, *N*_0_, *N*_1_, and *N*_2_ represent the number of turns for the primary winding and the two secondary windings, respectively. *i*_a_, *i*_b_, and *i*_c_ denote the winding currents of the primary Y-connected winding; *i*_a1_, *i*_b1_, and *i*_c1_ represent the winding currents shifted by −15°; and *i*_a2_, *i*_b2_, and *i*_c2_ represent the winding currents shifted by +15°. The three windings on the primary side are independent of each other, while the secondary windings are connected in a zigzag manner.

**Figure 12:**
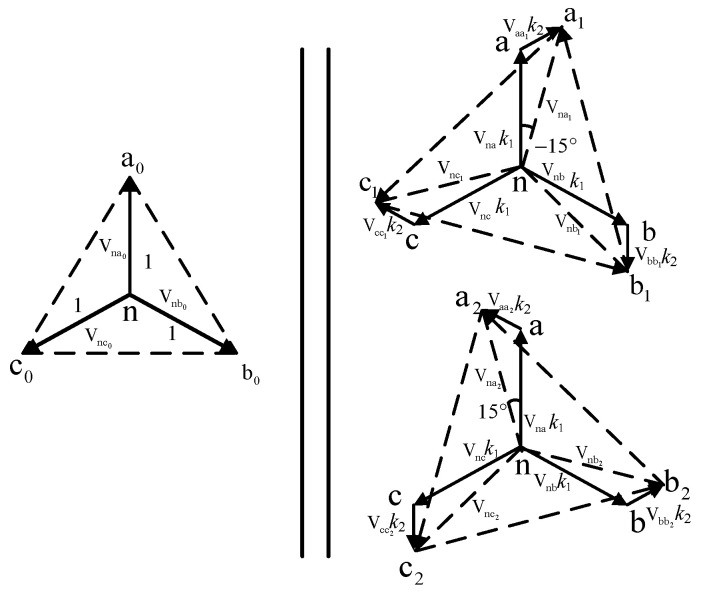
Zigzag transformer voltage vector diagram.

Figure 12 depicts the phasor diagram of the zigzag phase-shifting transformer. Here, *k*_1_ and *k*_2_ represent the balance achieved in the secondary leakage inductances due to the equal turns ratio per phase winding in the zigzag arrangement. To deliver two sets of three-phase voltages with a net phase difference of 30° to the three-phase rectifiers, assuming the input/output voltage magnitude of the original secondary side is 1, the star-connected winding voltage magnitude is *k*_1_, and the zigzag-connected winding voltage magnitude is *k*_2_. To achieve a phase difference of 30°, combining Figure 12 and the phase relationships, Equation (10) must be satisfied.

(10) $$\frac{1}{\sin105{}^{\circ}}=\frac{k_{1}}{\sin15{}^{\circ}}=\frac{k_{2}}{\sin60{}^{\circ}}$$

From Equation (10), the voltage magnitudes *k*_1_ and *k*_2_ can be obtained. Combining this with Figure 12, the turns ratio *K* of the phase-shifting transformer and the ratio of its winding turns should satisfy the following:

(11) $$K=\frac{\sqrt{6}N_{1}}{2N_{0}}=\frac{(3\sqrt{2}+\sqrt{6})N_{2}}{2N_{0}}$$

From Equations (10) and (11), it is evident that based on the phase-shifting angle requirements of the rectifiers for the phase-shifting transformer, the magnitudes of *k*_1_ and *k*_2_ can be calculated. Additionally, following the requirements for the step-up or step-down voltage transformation by the phase-shifting transformer, the turns ratio *K* between the primary and secondary sides can be determined.

### 2.4. Dual-Tap Converter

**Figure 13:**
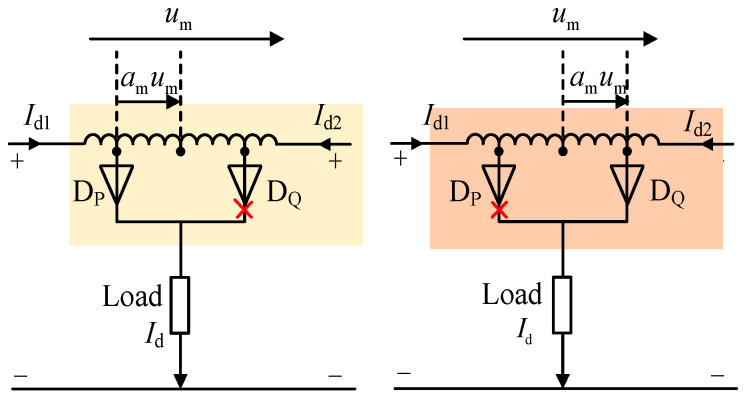
Double-tap operating modes.

According to the polarity of the terminal voltage *u*_m_ of the dual-tap converter in Figure 13, there are two operating modes for the dual-tap converter, as shown in Figure 13. Here, *a*_m_ represents the transformation ratio of the tap converter, *i*_d1,_ and *i*_d2_ are the output currents from the two sets of three-phase rectifier bridges, *I*_d_ is the load current, and D_P_ and D_Q_ are the two diodes of the dual taps.

Operating mode I: When *u*_m_ > 0, diode D_P_ conducts. In this mode, the output currents from the two sets of three-phase rectifier bridges satisfy the following:

(12) $$\left\{ \begin{array}{l} i_{d1}=0.5i_{d}+a_{m}i_{d}\\ i_{d2}=0.5i_{d}-a_{m}i_{d} \end{array}\right.$$

Operating mode II: When *u*_m_ < 0, diode D_Q_ conducts. In this mode, the output currents from the two sets of three-phase rectifier bridges satisfy the following:

(13) $$\left\{ \begin{array}{l} i_{d1}=0.5i_{d}-a_{m}i_{d}\\ i_{d2}=0.5i_{d}+a_{m}i_{d} \end{array}\right.$$

From the Equations (12) and (13), it can be observed that with the use of a dual-tap converter, the output current of the three-phase rectifier bridge consists of two components. The first component is 0.5*i*_d_, representing the load current of the proposed MPR. The second component is *a*_m_*i*_d_, representing the circulating current flowing between the tap converter and the rectifier bridge. When this circulating current meets certain conditions, it effectively suppresses harmonic currents in the rectifier. With the use of a dual-tap converter, the input current of the proposed MPR also consists of two parts: One part is the input current of the 12-pulse rectifier, and the other part is the manifestation of the DC-side circulating current on the AC side.

Based on the operating modes of the dual-tap converter, the switch functions *S*_P_ and *S*_Q_ of diodes D_P_ and D_Q_ can be expressed as follows:

(14) $$\begin{array}{l} S_{p}=\left\{ \begin{array}{l} 0\;\varphi \omega t\in \left[\frac{k\pi }{3},\frac{\pi }{6}+\frac{k\pi }{3}\right)\\ 1\;\varphi \omega t\in \left[\frac{\pi }{6}+\frac{k\pi }{3},\frac{(k+1)\pi }{3}\right) \end{array}\right.\\ S_{q}=\left\{ \begin{array}{l} 1\;\varphi \omega t\in \left[\frac{k\pi }{3},\frac{\pi }{6}+\frac{k\pi }{3}\right)\\ 0\;\varphi \omega t\in \left[\frac{\pi }{6}+\frac{k\pi }{3},\frac{(k+1)\pi }{3}\right) \end{array}\right. \end{array}$$

## 3. Optimal Design of Turns Ratio for Dual-Tap Transformer

Figure 12 shows that the three single-phase windings on the primary side of the zigzag isolation transformer are independent of each other. In comparison, the two windings on the secondary side are connected in a manner shifted by positive and negative 15 degrees. Based on the connection form and turns ratio relationship of the high-frequency phase-shifting transformer, it can be inferred that its primary side voltage *u*_a1_, *u*_b1_, and *u*_c1_ satisfies the following:

(15) $$\left\{ \begin{array}{l} u_{a1}=Ku_{\mathrm{apri}}∠15^{{}^{\circ}}\;,u_{a2}=Ku_{\mathrm{apri}}∠-15^{{}^{\circ}}\\ u_{b1}=Ku_{\mathrm{bpri}}∠15^{{}^{\circ}}\;,u_{b2}=Ku_{\mathrm{apri}}∠-15^{{}^{\circ}}\\ u_{c1}=Ku_{\mathrm{cpri}}∠15^{{}^{\circ}}\;,u_{c2}=Ku_{\mathrm{apri}}∠-15^{{}^{\circ}}\; \end{array}\right.$$

In Equation (15), *K* represents the turn ratio between the primary and secondary sides. Based on the voltage relationship of the secondary windings of the high-frequency phase-shifting transformer, the switching functions of the three arms *S*_a1_, *S*_a2_, and *S*_a3_ of Rec I can be derived as given:

(16) $$\left\{ \begin{array}{l} S_{a1}(t)=\frac{1}{2}\left\{\mathrm{sgn}\left[u_{a1}(t)-u_{c1}(t)\right]-\mathrm{sgn}\left[u_{b1}(t)-u_{a1}(t)\right]\right\}\\ S_{b1}(t)=\frac{1}{2}\left\{\mathrm{sgn}\left[u_{b1}(t)-u_{a1}(t)\right]-\mathrm{sgn}\left[u_{c1}(t)-u_{b1}(t)\right]\right\}\\ S_{c1}(t)=\frac{1}{2}\left\{\mathrm{sgn}\left[u_{c1}(t)-u_{b1}(t)\right]-\mathrm{sgn}\left[u_{a1}(t)-u_{c1}(t)\right]\right\} \end{array}\right.$$

**Figure 14:**
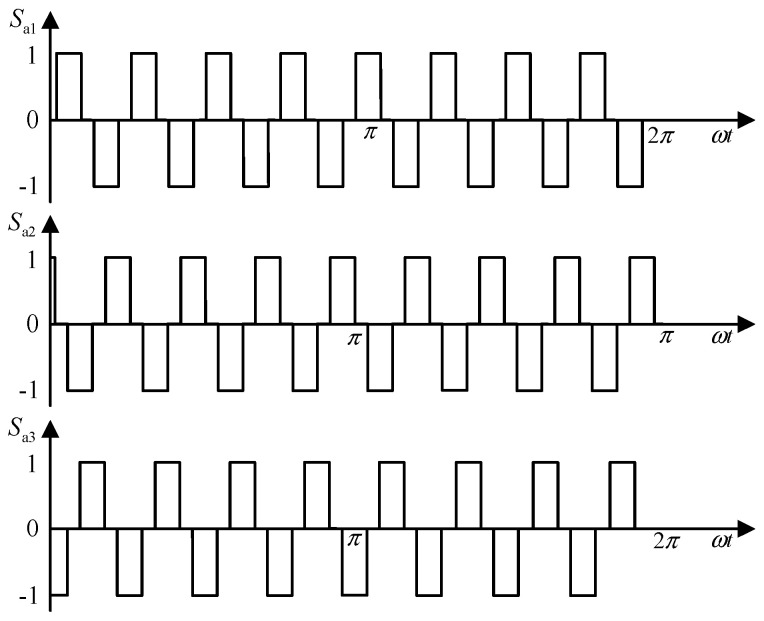
Switching function of *S*_a1_ in Rec I.

Figure 1 depicts the switching functions of Rec I in Figure 14. Similarly, the waveform of the switching functions for Rec II can be obtained.

According to Figure 14, after replacing the line-frequency phase-shifting transformer with the proposed three-stage PEPT, the operating mode of the rectifier bridge remains unchanged, but the operating frequency is significantly increased. Due to the modulation effect of the dual tap, the output currents *i*_d1_ and *i*_d2_ of Rec I and Rec II can be expressed as follows:

(17) $$\left\{ \begin{array}{l} i_{d1}=0.5i_{d}+(S_{p}-S_{q})a_{m}i_{d}\\ i_{d2}=0.5i_{d}-(S_{p}-S_{q})a_{m}i_{d} \end{array}\right.$$

In Equation (17), *i*_d_ represents the effective value of the load current. Using the switching function method, the output current of the phase-shifting transformer can be obtained by the following:

(18) $$\left\{ \begin{array}{l} i_{a1}=S_{a1}i_{d1};i_{b1}=S_{a1}i_{d1};i_{c1}=S_{c1}i_{d1}\\ i_{a2}=S_{a2}i_{d2};i_{b2}=S_{b2}i_{d2};i_{c2}=S_{c2}i_{d2} \end{array}\right.$$

Based on the zigzag-type isolation transformer winding structure shown in Figure 11 and applying Kirchhoff’s current law and the ampere-turn balance principle, the following can be derived:

(19) $$\left\{ \begin{array}{l} N_{0}i_{a}+N_{2}i_{c1}+N_{2}i_{b2}=N_{1}i_{a1}+N_{1}i_{a2}\\ N_{0}i_{b}+N_{2}i_{a1}+N_{2}i_{c2}=N_{1}i_{b1}+N_{1}i_{b2}\\ N_{0}i_{c}+N_{2}i_{b1}+N_{2}i_{a2}=N_{1}i_{c1}+N_{1}i_{c2} \end{array}\right.$$

By combining Equations (15)–(19), it can be derived that the input current *i*_apri_ satisfies the following:

(20) $$i_{\mathrm{apri}}=I_{d}\{\left[\frac{1}{2}+(S_{p}-S_{q})a_{m}\right](0.816S_{a1}-0.299S_{c1})+\left[\frac{1}{2}-(S_{p}-S_{q})a_{m}\right](0.816S_{a2}-0.299S_{b2})$$

According to the current symmetry, *i*_apri_ in [0, *π*/16] can be expressed:

(21) $$i_{\mathrm{apri}}=\left\{ \begin{array}{ll} 0.598a_{m}I_{d} & \omega t\in \left[0,\frac{\pi }{96}\right)\\ (0.5575-0.517a_{m})I_{d} & \omega t\in \left[\frac{\pi }{96},\frac{\pi }{48}\right)\\ (0.5575+0.517a_{m})I_{d} & \omega t\in \left[\frac{\pi }{48},\frac{\pi }{32}\right)\\ (0.9655-0.299a_{m})I_{d} & \omega t\in \left[\frac{\pi }{32},\frac{\pi }{24}\right)\\ (0.9655+0.299a_{m})I_{d} & \omega t\in \left[\frac{\pi }{24},\frac{5\pi }{96}\right)\\ 1.115a_{m}I_{d} & \omega t\in \left[\frac{5\pi }{96},\frac{\pi }{16}\right) \end{array}\right.$$

**Figure 15:**
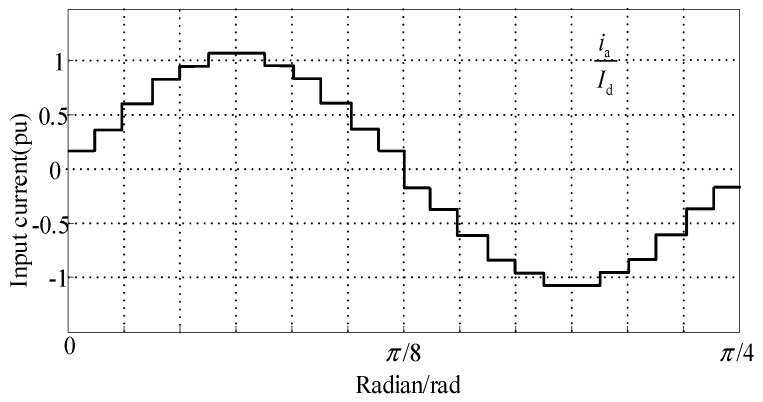
Waveform of a-phase input line current.

The corresponding waveform of the current value *i*_apri_ for the remaining three-quarters of the period is depicted in Figure 15.

The effective value *i*_rms_ of the current is given below:

(22) $$i_{\mathrm{rms}}=\sqrt{\frac{1}{\pi }\int_{-\pi /2}^{\pi /2}f^{2}(x)dx}$$

Combining Equation (22) and Figure 15, the input current RMS *I*_rms_ is obtained:

(23) $$I_{\mathrm{rms}}=I_{d}\sqrt{0.178a_{m}^{2}+0.622}$$

By utilizing odd extension for the Fourier series decomposition, the effective value of the fundamental component, *I*_af_ can be obtained as follows:

(24) $$I_{\mathrm{af}}=I_{d}(0.699+0.0493a_{m})$$

The following is the formula for calculating the total harmonic distortion (THD):

(25) $$\mathrm{THD}=\frac{\sqrt{I_{\mathrm{rms}}^{2}-I_{\mathrm{af}}^{2}}}{I_{\mathrm{af}}}$$

**Figure 16:**
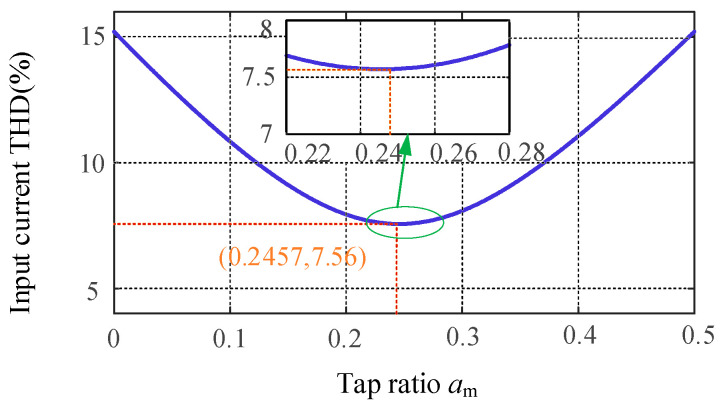
Relationship between AC side input voltage THD and tap turns ratio *a*_m_.

Substituting Equations (23) and (24) into Equation (25), the relationship between the tap ratio *a*_m_ and the THD of the input current can be depicted as shown in Figure 16. Differentiating it yields a minimum THD value of 7.56% when *a*_m_ equals 0.2457.

## 4. Test Validation and Analysis

**Figure 17:**
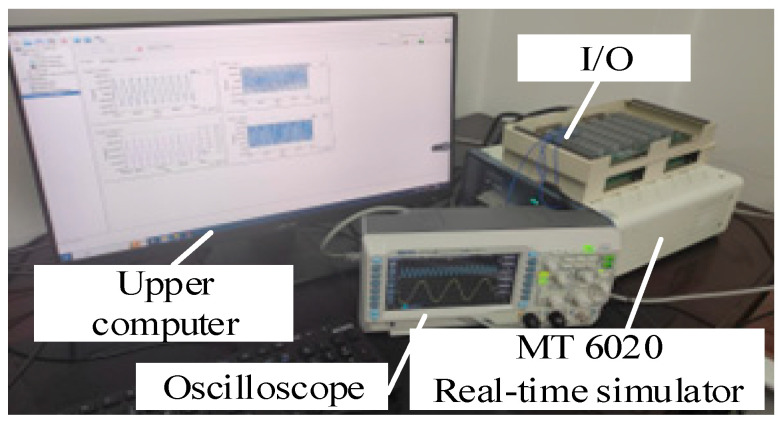
Hardware-in-the-loop real-time test platform.

To validate the correctness and effectiveness of the theoretical analysis mentioned above, we constructed the proposed rectifier model using the Starsim semi-physical testing platform developed by Shanghai Yuankuan Energy’s Starsim HIL real-time simulation software 5.0 and HIL real-time simulator (Modeling Tech, Shanghai, China). As shown in Figure 17, real-time semi-physical validation was conducted on the testing system (MT6020) with a sampling frequency of 20 kHz and a step size of 5 μs. The main parameters of the rectifier are shown in Table 1.

**Figure 18:**
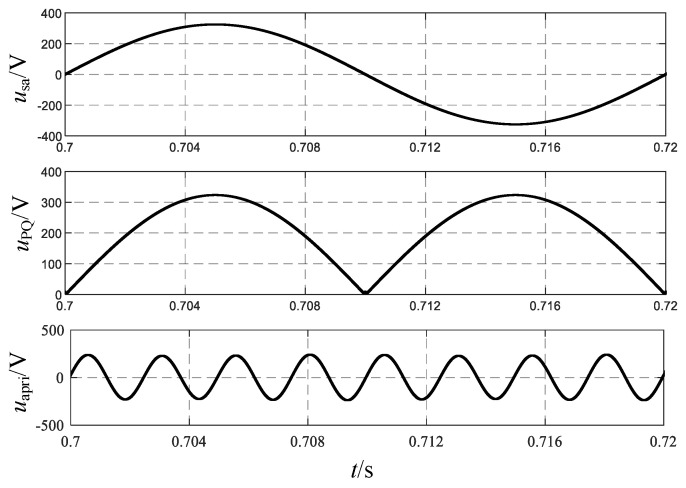
Output voltage of each part of the three-stage PEPT for the proposed MPR.

**Figure 19:**
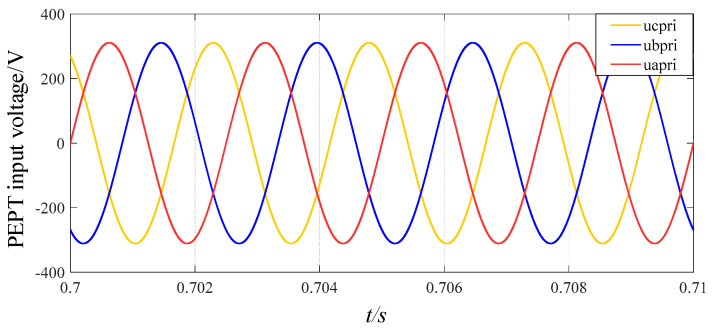
Three input voltages of the three-stage PEPT for the proposed MPR.

Taking phase a as an example, Figure 18 shows the output voltage waveforms of each part of the three-stage PEPT for the proposed shunt zigzag double-tap low-harmonic MPR. The frequency of the input voltage on the primary side of the transformer is increased from 50 Hz to 400 Hz, consistent with the theoretical analysis. Figure 19 depicts three input voltages of the phase-shifting transformer as a 400 Hz sinusoidal AC side.

**Figure 20:**
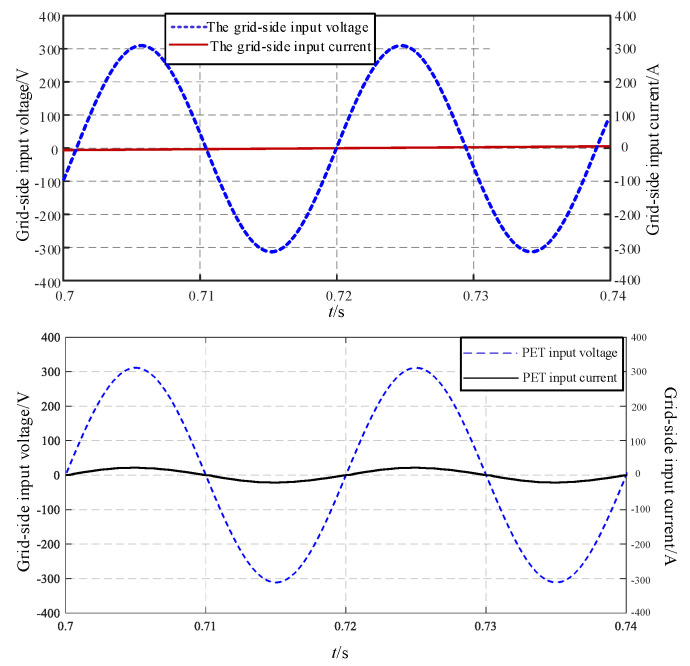
The input voltage and current of the three-stage PEPT.

Figure 20 displays the waveforms of the input voltage and input current of the three-stage PEPT. It is evident from Figure 20 that the voltage and current are in phase, achieving the purpose of power factor correction.

**Figure 21:**
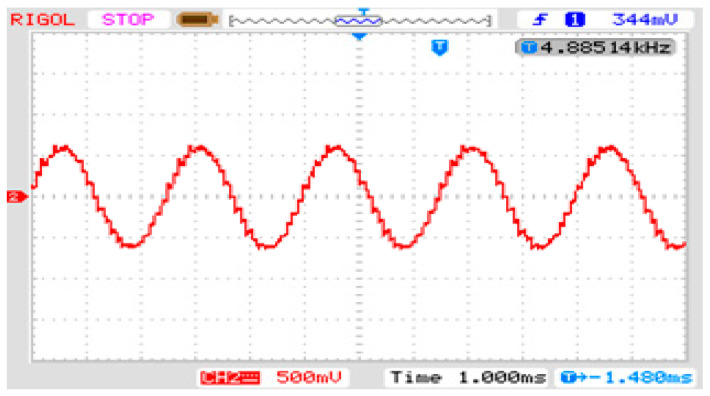
The corresponding test result of the primary input line current of the PEPT.

Figure 21 depicts the waveform of the grid-side input line current, exhibiting eight sets of 24-step waveforms within one cycle. It is evident from the figure that the input line current exhibits certain peaks and is not entirely flat. This phenomenon is attributed to the leakage inductance of the transformer and the utilization of hard switching in the circuit.

**Figure 22:**
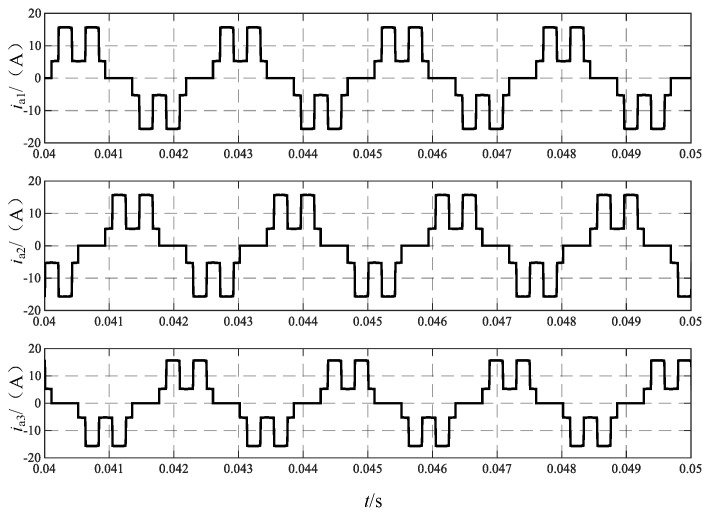
Transformer primary input current *i*_a1_, *i*_a2_, and *i*_a3_.

Figure 22 illustrates the waveform of the input current to Rec I, with currents *i*_a1_, *i*_b1_, and *i*_c1_ depicted from top to bottom. The operation of the dual tap on the DC side results in the output current of the phase-shifting transformer becoming a two-level current.

**Figure 23:**
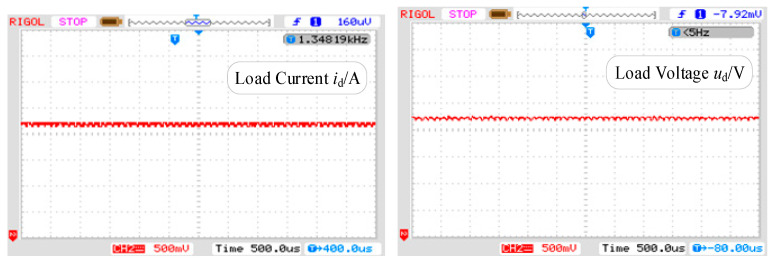
The corresponding test results of the load current *i*_d_ and the voltage *u*_d_.

As shown in Figure 23, the waveform of the load voltage/current test exhibits numerous spikes in both voltage and current due to the high-frequency switching of the switching devices during the experimental process, thus validating the theoretical derivation.

**Figure 24:**
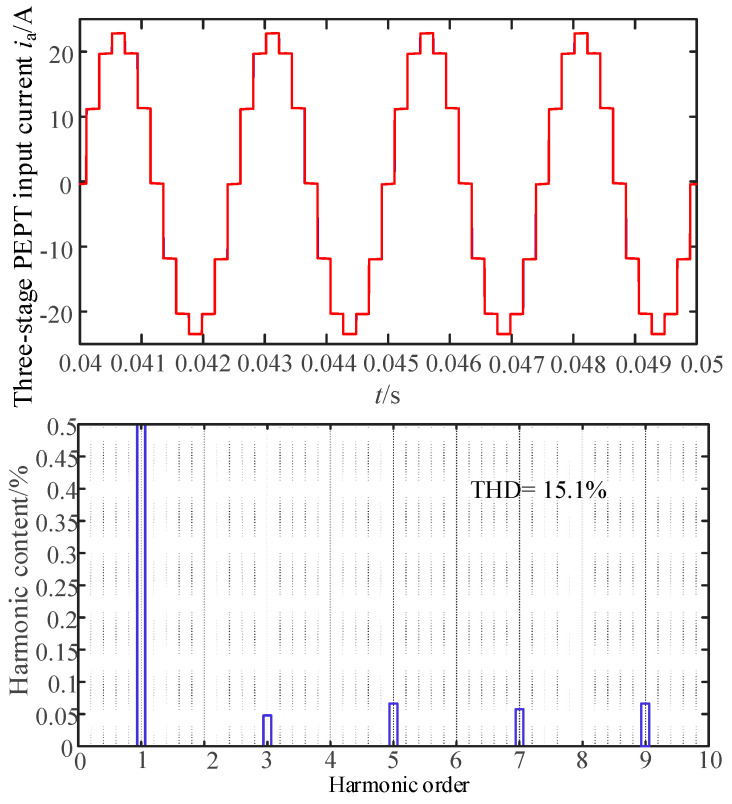
The results of the input current *i*_a_ and the FFT analysis without double-tap converter.

**Figure 25:**
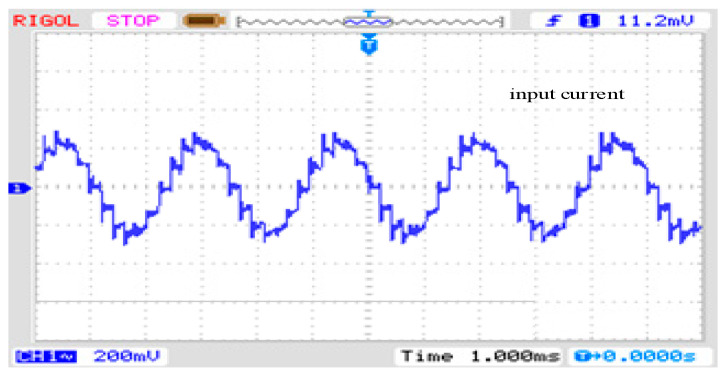
The corresponding test result of the input current *i*_a_ without a double-tap converter.

In addition to the PFC filtering on the grid side of the entire system, to discuss the harmonic mitigation effect of the dual-tap transformer, this study also conducted tests and discussions with and without the dual-tap transformer. As shown in Figure 24, when there is no dual-tap transformer, the input current of the phase-shifting transformer exhibits a 12-pulse waveform with a THD value of 15.1%, consistent with the conclusion in reference [1]. Figure 25 shows the test waveform.

**Figure 26:**
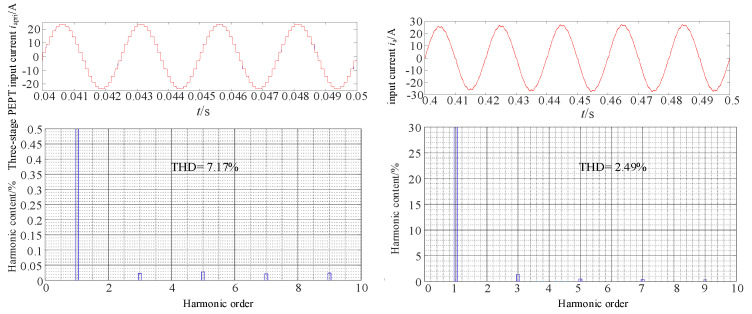
The results of the input current *i*_apri_ and the FFT analysis with a double-tap converter.

Figure 26 presents the system input current and the FFT analysis results after incorporating the dual-tap transformer. Compared to Figure 24, the test results in Figure 26 show that with the inclusion of the dual-tap transformer, the THD value of the input current to the phase-shifting transformer decreases to 7.17%. After passing through the PFC converter, the system input current approaches a sinusoidal waveform, decreasing from 7.17% to 2.49%, meeting harmonic standards. Overall, this proposed topology achieves both MPR size reduction and harmonic mitigation.

In addition, Table 2 shows that the single passive harmonic suppression circuit reduces the THD value of the input current but still does not meet the IEEE 519 standard [26]. The double passive harmonic suppression and hybrid harmonic suppression methods reduce the THD value to below 6%, satisfying most applications. The proposed zigzag double-tap MPR based on a three-stage PEPT in this paper reduces the corresponding THD value of the input current to 2.49%, meeting the requirements for high-power applications with stricter demands on power quality.

## 5. Adaptability Analysis of Proposed MPR

To investigate whether the power quality of the proposed zigzag double-tap low-harmonic MPR based on the three-stage PEPT is affected by different loads, the influence of different resistance values under pure resistive loads was first tested. It was assumed that the light load operation of the load is 100 Ω, and the full load operation is 20 Ω. The data obtained from the variation of the load from full load to light load are shown in Table 3.

From Table 3, it can be observed that during the transition of the rectifier load from 20 Ω to 100 Ω, the THD of the grid-side input current remains below 3%. Therefore, it can be inferred that the proposed three-stage PEPT MPR circuit maintains normal input current quality when the system load changes. This indicates its capability to effectively meet the specific requirements of power quality in demanding applications.

In addition, high-power rectifiers used in industrial settings face diverse types of loads. Ideally, if we consider load types without inductance, they can be categorized into two types: *R* and *RC*. However, in practical rectifiers, there are often magnetic devices involved, which inevitably introduce leakage inductance during operation. This results in the output load types becoming *RL* and *RLC*. The load parameters are sequentially set as *R* (20 Ω), *RC* (20 Ω, 4700 μF), *RL* (20 Ω, 50 mH), and *RLC* (20 Ω, 50 mH, and 4700 μF). The adaptability test results obtained are shown in Table 4.

From Table 3, it can be observed that under load types of *RC*, *RL*, or *RLC*, all power quality parameters remain normal, with THD values consistently below 3%, thus complying with harmonic standards. Therefore, the designed three-level PET MPR is applicable under most load conditions.

## 6. Conclusions

This paper aimed to reduce the size of traditional phase-shifting transformers while improving the harmonic distortion of the grid-side input current of power electronic MPR. A novel shunt zigzag double-tap low-harmonic MPR based on a three-stage PEPT is proposed. Through theoretical derivation and experimental verification, we achieved the following:

(1) Reducing the size of conventional industrial frequency phase-shifter transformers to be suitable for applications where the size of the transformer is strictly required;
(2) Weakening the harmonic distortion rate of grid-side input current to meet harmonic standards;
(3) Improving the function of power factor correction and ensuring the input voltage and current are kept in the same phase;
(4) Investigating the proposed MPR load adaptability to determine the harmonic distortion extent so that it is very suitable for high-power applications.

## Figures and Tables

**Table 1:** The main parameters of rectifier.

Circuit Parameters/Symbols	Numerical Value
The effective value of the input phase voltage/*U*_m_	220 V
PFC converter inductor/*L*_a_	0.3 mH
PFC converter capacitor/*C*_a_	2000 μF
SPWM inverter inductor/*L*_as_	1 mH
SPWM inverter capacitor/*C*_as_	10 μF
Grid-side voltage frequency/*f*_v_	50 Hz
Phase-shifting transformer operating frequency/*f*	400 Hz
Phase-shifting transformer turns ratio/*N*_0_:*N*_1_:*N*_2_	$1:\sqrt{6}/3:(3\sqrt{2}-\sqrt{6})/6$
Load resistance/*R*	20 Ω
Load filtering inductor/*L*	15 mH

**Table 2:** Comparison of different harmonic suppression schemes.

Topology	Input Current THD/%	Pulse Number
Two-stage PET [15]	16%	12
Single passive MPR [27]	7.52%	24
Two-stage PET double passive [28]	4.65%	24
Two-stage PET single passive [28]	7.44%	24
Double-star uncontrolled PET [29]	31%	6
Three-stage PEPT in this paper	2.49%	24

**Table 3:** Power Quality Parameters under Different Load Resistance Values.

Load/Ω	*i*_a_’s THD/%	*U*_d_/V
20	2.49	513.3
40	2.46	513.8
60	2.54	514
80	2.60	514.1
100	2.64	514.4

**Table 4:** Power Quality Parameters under Different Load Types.

Load Types	*i*_a_’s THD/%	*U*_d_/V
RL	2.48	513.1
RC	2.91	515
RLC	2.93	514.8

## Data Availability

The raw data supporting the conclusions of this article will be made available by the authors on request.
